# Splenic Infarct Secondary to High Altitude Exposure in Sickle Cell Trait Patients: A Case Series

**DOI:** 10.7759/cureus.9815

**Published:** 2020-08-17

**Authors:** Luis Gonzalez, Andres F Shapiro, Alfonso Tafur, Carlos Plaza-Meneses, Brenner Sabando

**Affiliations:** 1 Medicine, Nassau University Medical Center, East Meadow, USA; 2 Hematology, Hospital Luis Vernaza, Guayaquil, ECU; 3 Medicine, Hospital Luis Vernaza, Guayaquil, ECU; 4 Hematology, Hospital Luis Vernaza/Universidad Espíritu Santo (UEES), Guayaquil, ECU

**Keywords:** splenic infarct, sickle cell trait, high altitude

## Abstract

The sickle cell trait is considered a benign entity that generally does not show clinical manifestations. However, some complications have been described under certain conditions, such as a decrease in oxygen level, dehydration, and strenuous physical efforts. Among them, splenic infarct is a rare complication that presents as left upper abdominal pain in a situation of stress such as high altitude exposure. We present two cases of splenic infarcts in patients with undiagnosed sickle cell trait who showed to our institution with severe abdominal pain after coming from high altitude cities.

## Introduction

The sickle cell trait (SCT) is found in individuals with an inherited hemoglobin S (HbS) gene and a normal one hemoglobin gene [1]. The HbS is a result of glutamic acid to valine substitution in position six of the β-globin gene on chromosome 11 [2]. In 2010, the incidence of SCT in the U.S. was 15.5 per 1,000 newborns, with 73.1 for the black population and 6.9 per 1,000 newborns for Hispanics [3]. The amount of HbS distributed follows a trimodal pattern depending on the amount of α-globin chains (40%, 35%, 20-25%) [4]. There is an increased chance of polymerization and sickling with more amount of HbS [4].

Before, it was considered as a benign condition with protection to malaria and no painful episodes. However, several complications have been described in the literature as case reports, including venous thromboembolism, renal involvement, and splenic infarcts [5]. There are less than 100 reported cases of splenic infarcts in SCT associated with high altitude exposition [6,7]. Here, we present two unrelated cases of previously healthy young males who presented to a hospital located in Guayaquil, Ecuador, at sea level, after visiting high altitude cities. They were diagnosed with splenic infarcts related to undiagnosed SCT.

## Case presentation

Case 1

**Figure 1:**
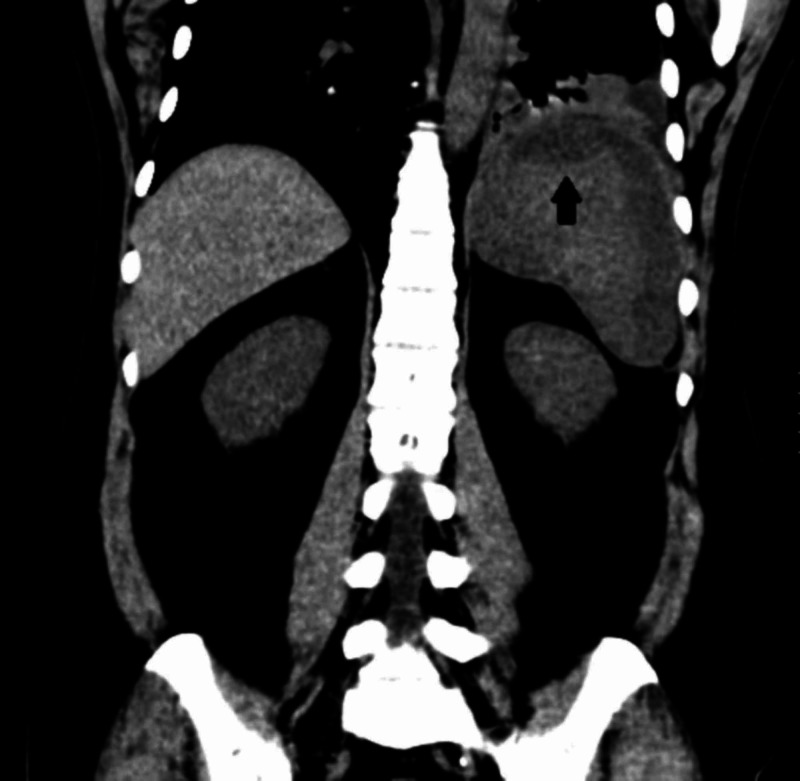
CT of the abdomen Coronal CT of the abdomen showing splenomegaly and low attenuated images in the spleen compatible with splenic infarction.

A 25-year-old Hispanic male with no known past medical history presented to the hospital complaining of left upper abdominal quadrant pain. He stated that pain started after he returned from a trip to a location at 16,700 feet of altitude. In the hospital, his vital signs were within normal limits except for tachycardia. On the physical exam, there was splenomegaly with tenderness to palpation. The ultrasound showed heterogeneous images in the spleen compatible with ischemic lesions, which were confirmed later as splenic infarcts with computed tomography (CT) of the abdomen (Figure 1). His lab results were as follows: hemoglobin of 15 g/dL, hematocrit of 49%, leukocytes of 6.28 x 10^9^/L, and platelets of 453 x 10^9^/L. Trying to find the cause, we ordered protein C, protein S, antithrombin III, factor V Leiden, and D-dimer tests, which came back negative. After that, we conducted hemoglobin electrophoresis, showing hemoglobin A (HbA) of 57.5%, HbS of 39%, hemoglobin A2 (HbA2) of 3.5%, and hemoglobin F (HbF) 1.9%. The patient was diagnosed with SCT, which was treated with hydration and pain management until symptoms resolved.

Case 2

**Figure 2:**
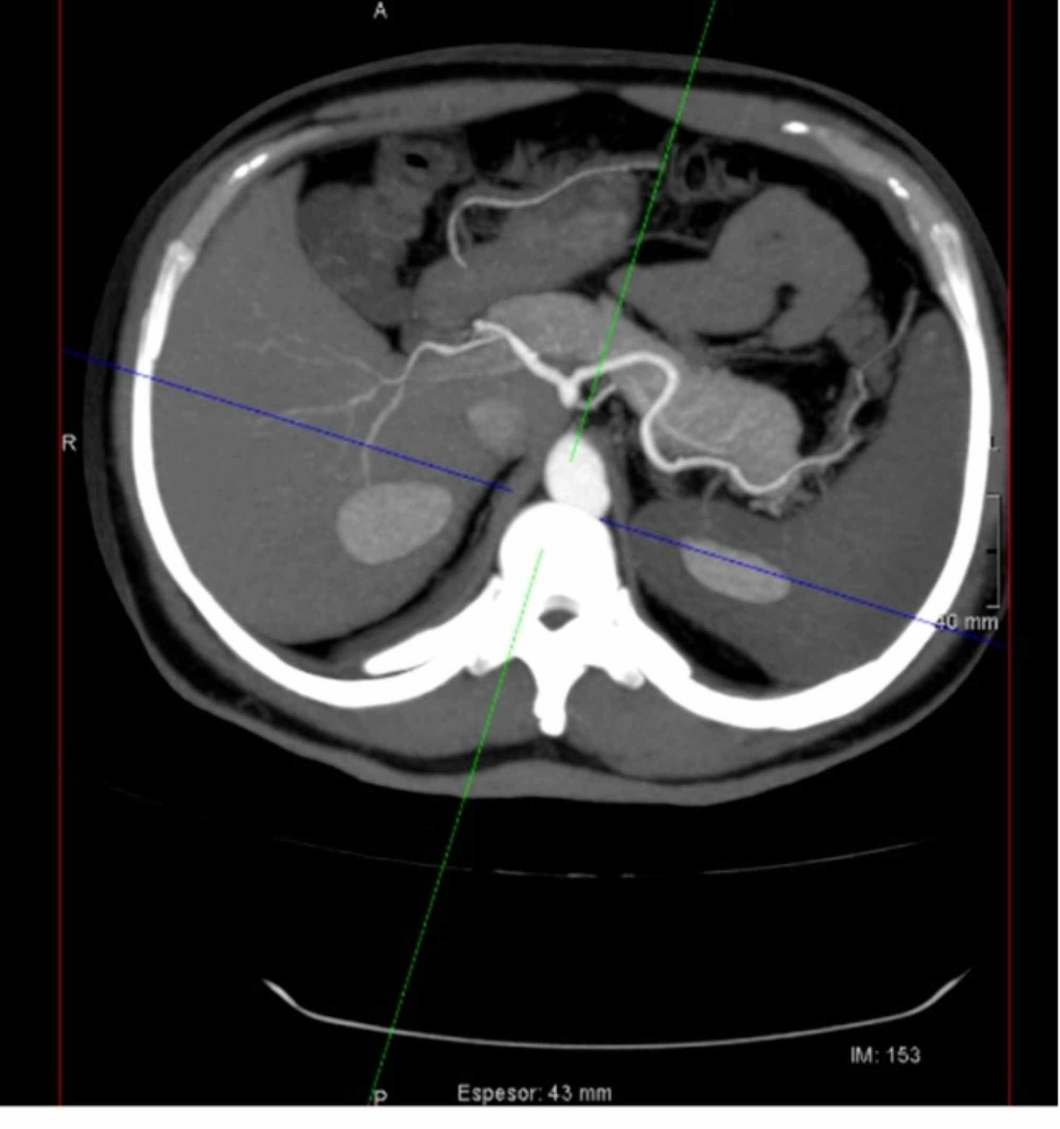
Axial contrast-enhanced CT of the abdomen Axial contrast-enhanced CT of the abdomen showing splenomegaly and lack of spleen enhancement suggesting splenic infarct.

A 20-year-old Hispanic male with no known past medical history presented to our hospital complaining of left upper abdominal quadrant pain and jaundice. These manifestations presented after he went to a city located at an altitude of 10,341 feet. On the physical exam, the patient showed splenomegaly, which was tender to palpation. In the ultrasound, there were multiple heterogeneous images within the spleen. In the CT angiogram of the abdomen, there was notable splenomegaly and lack of enhancement of the spleen compatible with splenic infarct (Figure 2). The complete blood count study showed hemoglobin of 13.8 g/dL, hematocrit of 38%, leukocytes of 13.3 x 10^9^/L, and platelets of 157 x 10^9^/L. His total bilirubin was 4.30 mg/dL, with indirect bilirubin of 3.49 mg/dL. Looking for a thrombogenic explanation, we studied protein C, protein S, antithrombin III, factor V Leiden, and D-dimer, but they were negative. Finally, the hemoglobin electrophoresis results showed HbA of 55%, HbS of 40%, HbA2 of 3.2%, and HbF of 1.8%, giving a diagnosis of SCT. During his admission, he was treated with conservative measures, such as symptom control and hydration, until he was discharged home.

## Discussion

We presented two patients without known previous pathology who had sudden abdominal pain after traveling to high altitudes cities. One of the cases also had jaundice. Both of them were diagnosed with splenic infarcts, and further investigations revealed that they were SCT carriers.

Splenic infarction is a rare and overlooked cause of abdominal pain [8]. It can be caused by thromboembolism, secondary to cardiovascular disease, or hypercoagulable state. Other causes are acute spleen enlargement secondary to infection or hematological diseases, obstruction of the splenic artery or vein, infiltrative diseases, and autoimmune disorders (e.g., polyarteritis nodosa, systemic lupus erythematosus) [9]. Most commonly, it presents as abdominal pain located in the left upper abdomen or sometimes in the epigastrium [8,10]. Diagnosis confirmation is made with contrast CT of the abdomen, which is more sensitive than the abdominal ultrasound [11]. They are seen as peripheral wedge areas that do not enhance with contrast [12].

SCT is one of the described causes of splenic infarction. In the literature, from 1985 to 2013, only 34 cases of high altitude-related splenic infarcts in the SCT population were reported [6]. These patients usually are unaware of their genetic condition until they develop symptoms sharply after they arrive at high altitude locations [1]. It is more prevalent in males and nonblack patients, and does not present in sickle cell disease due to their atrophic spleen secondary to repeated vaso-occlusive events [1]. Splenic infarct incidence directly correlates with the amount of HbS, especially in patients with an HbS higher than 40% [13]. As an interesting fact, people with SCT who were born in and live in high altitude cities do not present splenic infarcts likely due to an adaptation to low oxygen conditions since childhood [14].

Pathophysiology consists of HbS polymerization secondary to hypoxia, causing red cells to sickle and occlude the tributary vessels of the splenic artery [15]. The spleen is mainly affected due to previous splenomegaly secondary to splenic sequestration [1,16]. This leads to abdominal pain, with guarding of the abdominal wall musculature and rebound tenderness on the physical exam [6,17]. Patients can also present with nausea, vomiting, or dyspnea [17]. Dyspnea is caused by diaphragm irritation leading to decreased breathing or splinting [6].

Splenic infarction can lead to complications such as abscesses or pseudo-cyst formation, spleen rupture, and hemorrhage [16,18,19]. Generally it is benign, and conservative management is enough for relief of the symptoms. It consists of rest, hydration, oxygen support if needed, pain control, and descent to a lower altitude [20]. Indications for splenectomy are splenic rupture, abscess, sequestration crisis, and chronic pain despite supportive therapy [4,6]. In uncomplicated cases managed with conservative treatment, symptoms resolve between 7 and 14 days [16].

## Conclusions

Although splenic infarction associated with SCT is rare, this condition has to be kept in mind in patients who present with sudden abdominal pain after being exposed to high altitude. Once the splenic infarction is diagnosed, hemoglobin electrophoresis should be part of the workup to find the cause of the infarction. Finally, the management in the majority of cases consists of conservative therapy with symptomatic control and hydration.
